# The effect of vitamin D and magnesium supplementation on the mental health status of attention-deficit hyperactive children: a randomized controlled trial

**DOI:** 10.1186/s12887-021-02631-1

**Published:** 2021-04-17

**Authors:** Mostafa Hemamy, Naseh Pahlavani, Alireza Amanollahi, Sheikh Mohammed Shriful Islam, Jenna McVicar, Gholamreza Askari, Mahsa Malekahmadi

**Affiliations:** 1 Department of Community Nutrition, School of Nutrition and Food Sciences, Isfahan University of Medical Sciences, Isfahan, Iran; 2 Social Development and Health Promotion Research Center, Gonabad University of Medical Sciences, Gonabad, Iran; 3 Department of Epidemiology, School of Public Health and Safety, Shahid Beheshti University of Medical Sciences, Tehran, Iran; 4 Institute for Physical Activity and Nutrition (IPAN), School of Exercise and Nutrition Sciences, Deakin University, Melbourne, Australia; 5 Research Center for Gastroenterology and Liver Disease, Shahid Beheshti University of Medical Sciences, Tehran, Iran

**Keywords:** Attention-deficit hyperactivity disorder, Vitamin D, Magnesium, Supplementation, Randomized controlled trial

## Abstract

**Background:**

Attention-Deficit / Hyperactivity Disorder (ADHD) is a neurodevelopmental disorder, characterized by varying severity in attention deficit and hyperactivity. Studies have shown deficiencies in the serum level of magnesium and vitamin D in people with ADHD. The aim of this study is to determine the effect of vitamin D and magnesium supplementation on mental health in children with ADHD.

**Methods:**

We conducted a randomized, double blind, placebo-controlled clinical trial of 66 children with ADHD. Participants were randomly allocated to receive both vitamin D (50,000 IU/week) plus magnesium (6 mg/kg/day) supplements (*n* = 33) or placebos (n = 33) for 8-weeks. Strengths and difficulties questionnaire was used to evaluate children’s mental health at baseline and the end of the study.

**Results:**

After eight weeks of intervention, the serum levels of 25-hydroxy-vitamin D3 and magnesium increased significantly in the intervention group compared with the control group. Also, children receiving vitamin D plus magnesium showed a significant reduction in emotional problems (*p* = 0.001), conduct problems (*p* = 0.002), peer problems (*p* = 0.001), prosocial score (*p* = 0.007), total difficulties (*p* = 0.001), externalizing score (*p* = 0.001), and internalizing score (*p* = 0.001) compared with children treated with the placebo.

**Conclusion:**

Vitamin D (50,000 IU/week) and magnesium (6 mg/kg/day) co-supplementation for a duration of 8-weeks could improve the behavioral function and mental health of children with ADHD. However, further well-designed studies with a larger sample size are needed.

**Trial registration:**

IRCT2016030326886N1.

## Background

Attention-Deficit / Hyperactivity Disorder (ADHD) is characterized by varying severity in attention deficit and hyperactivity and is related to neurodevelopmental disorders. The prevalence of ADHD has been increasing over the past decades [1]. ADHD is commonly diagnosed in children with low academic and social development, learning disabilities, social dysfunction, low self-esteem, and impaired emotion regulation. Many of these symptoms persist from childhood and into adulthood, leading to a decreased quality of life [2–4]. Worldwide, 5 to 7% of school-age children have ADHD [5, 6]. In Iran, 5.03% of boys and 2.79% of girls of school age have been diagnosed with ADHD [7]. The exact cause of ADHD has not yet been identified, however, several environmental and genetic factors have been studied. For example, a genetic factor related to dopaminergic genotypes exposed to environmental factors, such as maternal smoking before birth, increases the risk of ADHD in children [8].

In general, the risk factors for ADHD include smoking, alcohol and substance abuse and maternal stress during pregnancy. Moreover low birth weight, preterm delivery, contaminants such as organophosphates, polychlorinated biphenyls, lead, artificial dye ores and low socio-economic status of the family [9] are also well known risk factors. Medications have a significant effect on improving the symptoms of ADHD, and psychological counseling reinforces this effect. However, despite treatments and counseling, a substantial number of children with ADHD remain symptomatic [10]. Due to this, many parents tend to seek “alternative” or “natural” therapies because of concerns about the side effects of medications. Previous studies have shown that approximately 24.7% of children with ADHD received some form of complementary and alternative medicine [11].

ADHD is a common neurodevelopmental disorder with associated vitamin and mineral deficiency. Nutrient deficiencies have not been shown to cause ADHD in children, but studies in children with ADHA have shown deficiencies in some nutrients such as magnesium and vitamin D [12, 13]. Evidence suggests that vitamin D plays a vital role in the proper functioning of the central nervous system (CNS) and mental health [14–16]. Although there is clear evidence that vitamin D levels are lower in children and adolescents with ADHD [17–19], the benefits of vitamin D supplementation in this group are still unclear. However, it is understood that the level of vitamin D within this population is lower compared to children without ADHD. It is recognised vitamin D plays a positive role in psychiatric diseases such as autism, depression and schizophrenia, therefore the aim of this study was to determine its role in children with ADHD [14, 20, 21]. Magnesium is another essential nutrient that is associated with cognitive impairment and can lead to symptoms such as fatigue, lack of concentration, nervousness and mood swings [22]. In a study by Baza et al., magnesium supplementation in patients with ADHD was shown to significantly improve the the clinical symptoms within the patients [23]. According to the results of one meta-analysis conducted by Effatpanah et al., children and adolescents with ADHD had significantly lower serum magnesium levels compared with the control group. However, it was suggested that further investigation is needed due to the heterogeneity between studies [13]. Research supports the role of vitamin D and in general. It seems that due to the role of vitamin D and magnesium in ADHD and the proven deficiency of these two substances within patients with ADHD, supplementation with these two micronutrients as a complementary treatment may be able to improve the symptoms of these patients as an effective adjunctive therapy. Therefore, we conducted this randomized controlled trial to assess the effect of vitamin D and magnesium co-supplementation on behavioral problems in children with ADHD [24]. This manuscript, in continuation of the mentioned study, intends to report the effect of supplementation with these micronutrients on children’s mental health.

## Methods

The present study is an 8-week double-blind, randomized controlled trial conducted based on the CONSORT guidelines [25]. During March to May 2016, 74 children with ADHD were recruited from the Clinic of Noor and Ali Asghar of Isfahan University of Medical Sciences. The sample size was calculated with 98% power (*n* = 33). Ethical approval was provided by the Ethics Committee of Isfahan University of Medical Sciences, Isfahan, Iran. The study is registered with the Iranian Registry of Clinical Trials with number; IRCT2016030326886N1.

Children aged between 6 and 12 years old, with a serum level of 25-hydroxyvitamin D_3_ less than 30 ng/dL [26], a diagnosis of ADHD based on the presence of at least 6 out of 9 cases of inattention and also at least 6 out of 9 cases of hyperactivity based on DSM IV (Diagnostic and Statistical Manual of Mental Disorders, fourth edition), and serum magnesium levels less than 2.3 mg/dL [27], and provided consent to participate in the study were included in the study. Participants were excluded if they were taking any multivitamin/mineral before initiating or during the study and suffering from any chronic medical or other psychiatric disorders.

Based on ethical considerations, the study method and our goals in this research were explained to each individual. After acquiring written consent from their parents or legal guardian, patients were divided into intervention or control group by randomized double block method after stratification by gender. An independent researcher made random allocation cards using computer-generated sequence and used sequentially numbered, sealed, opaque envelopes to conceal the allocation. Neither the researcher nor the participants were aware of the groups. The intervention group received a pearl of vitamin D (50,000 IU/week with lunch meal) and an oral tablet of magnesium (6 mg/kg/day with lunch meal) for a duration of 8-weeks [28]. Participants in the control group received a placebo, similar in appearance, color and taste to the two supplements (edible paraffin oil as a placebo for vitamin D, microcrystalline cellulose and stearic acid as a placebo for magnesium). Participant’s compliance was measured by comparing the serum levels of vitamin D and magnesium before, and after the intervention [29]. Also at the end of the study, we measured individuals’ compliance through the remaining supplements using the following formula: number of used tablets or pearls/ all given tablets or pearls × 100.

The baseline characteristics of individuals were collected by questionnaire. Height and weight were measured with a precision of 0.5 cm and 100 g respectively. Body mass index (BMI) was calculated by weight in kilograms divided by height in meters squared [30]. Serum levels of 25-OH-vitamin D and magnesium were assessed at baseline and the end of the intervention period, as explained in our previously published paper [24]. Serum levels of 25-OH-vitamin D were measured by enzyme-linked immunosorbent assay (ELISA) method with a commercially available ELISA kit from Immundiagnostik AG, Bensheim, Germany. Serum levels of magnesium were measured by an autoanalyzer (Hitachi 917, Roche Diagnostics® GmbH, Mannheim, Germany) using a commercially available kit.

The strength and difficulties questionnaire (SDQ) was used to evaluate the mental health status of participants as a primary outcome [31]. This questionnaire was completed by the childrens parents at baseline and the end of the study. This scoring system contains 25 questions. Each of the five scales of the SDQ is scored from 0 to 10, and one can add up four of these (emotional, hyperactivity, conduct, and peer problems) to create total difficulty score (range 0–40). We can combine emotional and peer items to obtain the internalizing problems score (range 0–20), and also acquire an externalizing score (range 0–20) with combining theconduct and hyperactivity questions.

We analyzed all data using SPSS software version 19 (SPSS, Inc., Chicago, IL, USA) and Stata version 14 (StataCorp LLC). We used the Kolmogorov–Smirnov test to examine the normal distribution of variables and equality of variances was checked by Leven’s test. Countinuse variable arereported by mean ± SD and categorical variables are reported by number (%). Chi-square and Mann-Whitney test were used to assess the significance level of general characterisitcs between the two groups. To assess the effect of intervention by adjusting sex, age, BMI and retalin dose univariate analysis (ANCOVA) was applied and adjusted mean was reported. Cohen’s d was used to estimate the effect size of the two independent groups, and partial Eta to the estimate effect size of the covariates in ANCOVA. A *p*-value of < 0.05 was considered as the significance level.

## Results

**Fig. 1:**
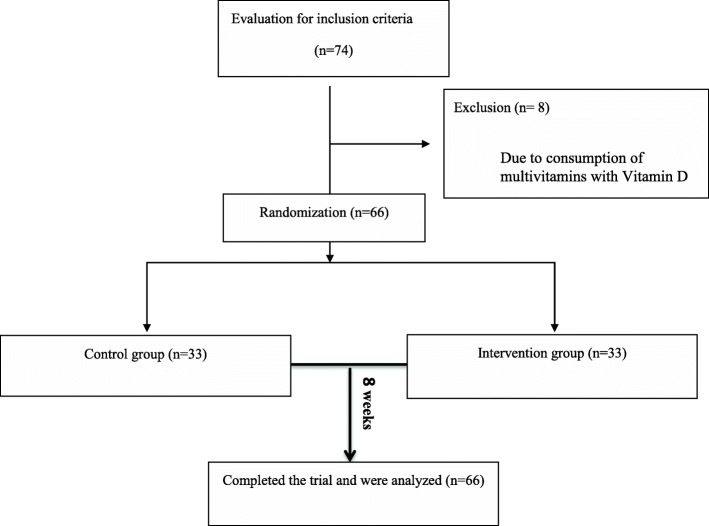
Participants flow diagram

Of the 74 children screened for this trial, 66 participants [intervention group (*n* = 33) and control group n = 33)] were selected based on the inclusion and exclusion criteria. From the 66 patients included at baseline, all participants completed the study (Fig. 1). In our study, more than 95% of supplements were consumed in both groups, and the rate of compliance was high. In the present study, we did not see any side effects from any of the supplements (vitamin D or magnesium).

**Fig. 2:**
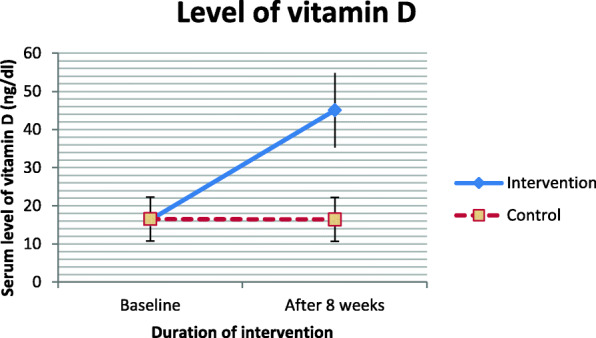
Serum 25-OH vitamin D levels of participants at study baseline and end of trial. *P* values obtained from independent samples t test. *P* value for comparison of changes between the two groups was 0.001

**Fig. 3:**
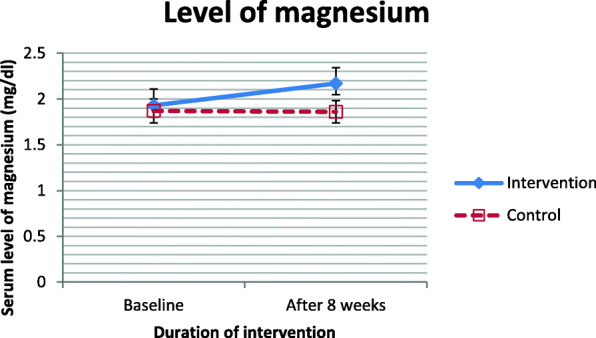
Serum magnesium levels of participants at study baseline and end of trial. *P* values obtained from independent samples t test. *P* value for comparison of changes between the two groups was 0.001

The mean age of the participants was 9.11 ± 1.61 years. The demographic and baseline data of the children are shown in Table 1. There was no statistically significant difference in age, weight, height, gender, and Ritalin dose between the two groups (Table 1). Participants were categorized based on BMI: underweight (BMI ≤ 19.5 kg/m^2^), normal weight (19.5 < BMI ≤ 25 kg/m^2^), over weight (25 < BMI ≤ 30 kg/m^2^) and obese (BMI>30 kg/m^2^). Moreover, the baseline levels of 25-OH-vitamin D and magnesium were not different between the intervention and control groups (Fig. 2 & 3). After the 8-week intervention, the serum levels of 25-OH-vitamin D and magnesium significantly increased in the intervention group compared to the control group (Fig. 2 & 3).

**Table 1:** General characteristics of the study participants^1^

	Intervention (*n* = 33)	Control (n = 33)	*P*
Age (years)	9.06 ± 1.76	9.15 ± 1.46	0.80
Weight (kg)	31.33 ± 9.93	31.17 ± 8.82	0.96
Height (cm)	129.46 ± 11.12	129.34 ± 9.48	0.87
BMI (kg/m^2^)			0.66
Underweight	3 (9.10%)	1 (3.00%)	
Normal weight	17 (51.50%)	21 (63.60%)	
Overweight	8 (24.20%)	7 (21.20%)	
Obese	5 (15.20%)	4 (12.10%)	
Sex			0.99
Boy (%)	23 (69.7%)	23 (69.7%)	
Girl (%)	10 (30.3%)	10 (30.3%)	
Ritalin dose (mg/kg)	31.33 ± 9.93	31.21 ± 8.81	0.93

^1^Data are presented as Means ± SD other than those specified

^2^ For comparison of numerical values mann-whitney test and for qualitative values chi-square test has been used

BMI, body mass index

The effect of co-supplementation of vitamin D and magnesium on the components of SDQ with and without adjusting for age, sex, BMI and Ritalin dose are shown in Table 2. The effect of intervention by adjustment on improvement emotional problem (*P* = 0.051), peer problem (*P* = 0.006), total difficulties (*P* = 0.009) and internalizing (*P* = 0.003) was significant. Peer ploblem was a significant effect on all covariates, externalizing was significant in all covariates except age and finally internalizing was significant in age and sex. The effect size of Cohen’s d showed that the mean differences, which are significant, also have larger effect effects. The effect sizes related to interventions in the precision of model for internalizing (14%), peer problem (12.2%) and total difficulties (11.2%) were more than others. Among the effect sizes written for the different models in Table 2, only the peer problem model was more accurate than the other models.

**Table 2:** The effect of vitamin D and magnesium supplementation compared with placebo on behavior in children with ADHD based on strengths and difficulties questionnaire^1^

Independent variables	Un adj.	Effect size (Cohen’s d)	Adj.		Effect size (Partial Eta)
	***p***-value		Covariate	p-value	
**Emotional problem**	0.025	- 0.565	**Sex** **Age** **BMI** **Ritalin dose** **Group**	0.181 0.681 0.811 0.877 0.051	0.031 0.003 0.001 0.001 0.063
**Conduct problem**	0.096	- 0.416	**Sex** **Age** **BMI** **Ritalin dose** **Group**	0.053 0.018 0.010 0.007 0.117	0.06 0.092 0.110 0.130 0.041
**Hyperactivity**	0.28	- 0.269	**Sex** **Age** **BMI** **Ritalin dose** **Group**	0.061 0.966 0.070 0.15 0.275	0.06 0.001 0.055 0.035 0.02
**Peer problem**	0.003	- 0.760	**Sex** **Age** **BMI** **Ritalin dose** **Group**	0.110 0.001 0.002 0.002 0.006	0.045 0.165 0.156 0.157 0.122
**Prosocial**	0.18	- 0.334	**Sex** **Age** **BMI** **Ritalin dose** **Group**	0.248 0.085 0.106 0.019 0.170	0.023 0.049 0.044 0.090 0.031
**Total difficulties**	0.005	- 0.712	**Sex** **Age** **BMI** **Ritalin dose** **Group**	0.017 0.045 0.018 0.013 0.009	0.093 0.067 0.091 0.100 0.112
**Externalizing**	0.105	- 0.404	**Sex** **Age** **BMI** **Ritalin dose** **Group**	0.026 0.151 0.011 0.013 0.120	0.081 0.035 0.110 0.109 0.042
**Internalizing**	0.001	- 0.855	**Sex** **Age** **BMI** **Ritalin dose** **Group**	0.052 0.041 0.141 0.070 0.003	0.061 0.070 0.036 0.054 0.140

For comparison of continus variables ANCOVA test has been used

BMI, body mass index

The adjusted mean for the SDQ component in gender and groups was summerised in Table 3. As indicated, the mean of the SDQ components in the intervention group is lower than the control group. Also, the mean of the SDQ components are less in girls compared to boys.

**Table 3:** The adjusted mean for the strengths and difficulties questionnaire components based ongender and groups

	group	Mean	SE	sex	Mean	SE
**Emotional problem**	**Intervention**	3.274	0.456	**boy**	4.362	0.356
	**Control**	4.555	0.455	**girl**	3.468	0.547
**Conduct problem**	**Intervention**	3.393	0.354	**boy**	4.286	0.276
	**Control**	4.186	0.352	**girl**	3.293	0.424
**Hyperactivity**	**Intervention**	6.162	0.337	**boy**	6.888	0.263
	**Control**	6.683	0.336	**girl**	5.957	0.404
**Peer problem**	**Intervention**	2.586	0.288	**boy**	3.513	0.225
	**Control**	3.748	0.287	**girl**	2.821	0.345
**Prosocial**	**Intervention**	7.132	0.388	**boy**	7.180	0.303
	**Control**	7.884	0.387	**girl**	7.836	0.465
**Total difficulties**	**Intervention**	15.415	0.984	**boy**	19.049	0.769
	**Control**	19.172	0.980	**girl**	15.538	1.179
**Externalizing**	**Intervention**	9.555	0.583	**boy**	11.174	0.455
	**Control**	10.869	0.581	**girl**	9.250	0.698
**Internalizing**	**Intervention**	5.860	0.562	**boy**	7.874	0.439
	**Control**	8.302	0.560	**girl**	6.289	0.673

*Covariates for age, BMI and Ritalin dose

## Discussion

To the best of our knowledge, the present study is one of the few studies to examine the effect of vitamin D, and magnesium supplementation in Iranian children with ADHD. The results showed that vitamin D and magnesium supplements could decrease emotional, peer problems, total difficulties and internalizing scores compared to placebo. However, these supplementations did not have a significant effect on conduct problem, prosocial, externlising and hyperactivity scores. Also, the intervention showed a lower mean for the SDQ components in girls compared to boys.

ADHD is a common psychiatric disorder among children influenced by genetics and environmental factors (for example, nutritional factors such as vitamin D and magnesium). ADHD has three symptoms, including impulsivity, hyperactivity, and lack of attention [32, 33]. Recent studies have shown that serum vitamin D levels in children with ADHD are significantly lower than children without ADHD [20, 34], and about 72% of children with ADHD have a magnesium deficiency [23]. A study by Elshorbagy et al. showed that vitamin D Supplementation in pateints with ADHD improved cognitive functions such as conceptual level, inattention, opposition, hyperactivity, and impulsivity domains compared to the control group, which confirm the findings of our study [34]. In a similar study, Mohammadpour et al. showed that vitamin D supplementation at a dose of 2000 units/day as adjunctive treatment to methylphenidate for eight weeks could improve evening symptoms in children with ADHD [35]. Another study found that vitamin D supplementation could prevent exacerbations and reduce impulsivity in people with ADHD in addition to improving behavioral problems [36]. Experimental studies in rats have shown that vitamin D deficiency impaired brain distortion, decreased release of neuronal growth factors, sensitivity to psychiatric stimulants (NMDA antagonist MK-801), and impaired attention processing [37]. Furthermore, vitamin D deficiency increased impulsivity as well as a lack of inhibitory control [38]. In addition, prenatal vitamin D deficiency leads to changes in genes associated with neuronal survival, speech and language, and dopamine synthesis [39]. Vitamin D regulates calcium transient in the brain and nerve growth by involving in migration and nerve growth, separation, neurotransmission, cell interaction and synaptic function. It also protects the neural system from reactive oxygen species, alters neuronal factors and monoamine levels, and regulates hormonal and serotonin pathways in the CNS [40]. Also, reducing the symptoms of patients with ADHD with vitamin D supplementation may be due to the association between vitamin D and dopamine levels in the brain [41]. Vitamin D arranges the Wnt/beta-catenin signaling pathway that has a role in early brain development by developing the expression of DKK-1, which inhibits the Wnt/beta-catenin signaling pathway [12]. Vitamin D is also involved in the synthesis of serotonin [12]. All the mechanisms can explain the reason for the improvement of parameters related to mental health with vitamin D supplementation in this study.

One study showed that serum magnesium levels in people with ADHD were significantly lower than in the general population as well as in laboratory references. Therefore, given the role of magnesium in the nervous system, it is likely that magnesium supplementation within this patient population can help improve symptoms [42, 43]. Similarly, magnesium supplementation has shown to improve several mental health parameters. A randomized clinical trial with magnesium supplementation (200 mg/day) for six months independent of other mental disorders coexisting with hyperactivity, significantly decreased hyperactivity compared to the control group and clinical state before supplementation [44]. The magnesium therapy led to improvements in the behavior, both large- and small-scale mobility, decreased the level of anxiety and aggression, increased the attention, corrected with the improvement of the magnesium homeostasis [45]. These results are consistent with our findings. Magnesium is involved in the pathogenesis of ADHD through various molecular mechanisms. Magnesium is an essential substance in the body and plays a vital role in biochemical and physiological neurological processes and controls the pathway of glutamate N-methyl aspartate,a stimulant that induces neurotoxicity [46]. Many studies have also shown that magnesium plays a crucial role in the conversion of essential fatty acids to long-chain omega-3 and omega-6 fatty acids as cofactors for desaturase enzymes [47]. Therefore, its deficiency causes brain dysfunction.

In a previously published manuscript from this study, children’s behavioral problems were examined with Conners’ Parent Rating Scale-48 (CPRS-48), the results of which indicated improved behavior [24]. In contrast, in the present manuscript, mental health was examined with SDQ tool, and the effects of improvement were proven for this outcome.

Our study had several limitations. First, we investigated the combined effect of magnesium and vitamin D, and the effect of each supplement was not studied separately. Second, our sample size was relatively small. Future studies should be designed to determine both the combined and individual effect of these micronutrients on people with ADHD in a larger population. Third, the dietary intakes of magnesium and vitamin D as essential cofounders were not assessed. Fourth, the duration of follow-up was short, so long-term effects are unknown. Finally, participants with low serum levels of these two micronutrients were included in the study, so it is unclear whether supplementation can be effective in patients with ADHD who are not deficient.

## Conclusion

Vitamin D (50,000 IU/week) and magnesium (6 mg/kg/day) co-supplementation for a duration of 8-weeks could improve the behavioral function and mental health in children with ADHD affected with low serum level of vitamin D and magnesium. However, further well-designed studies with larger sample sizes are needed to validate our findings and determine whether vitamin D and magnesium deficiency are the results of ADHD or whether they are risk factors for this disorder.

## Data Availability

The data that support the findings of this study are available from Nutrition department of Isfahan University of Medical Sciences but restrictions apply to the availability of this data, which were used under license for the current study, and are not publicly available. Data are however available from the authors upon reasonable request and with permission of Nutrition department of Isfahan University of Medical Sciences.

## Abbreviations

BMI: Body Mass Index
ADHD: Attention-Deficit / Hyperactivity Disorder
ELISA: Enzyme-Linked Immunosorbent Assay
SDQ: Strength and Difficulties Questionnaire
CNS: Central Nervous System
CPRS-48: Conners’ Parent Rating Scale-48
DSM IV: Diagnostic and Statistical Manual of Mental Disorders, fourth edition
