# Correction to: The effect of vitamin D and magnesium supplementation on the mental health status of attention-deficit hyperactive children: a randomized controlled trial

**DOI:** 10.1186/s12887-021-02683-3

**Published:** 2021-05-12

**Authors:** Mostafa Hemamy, Naseh Pahlavani, Alireza Amanollahi, Sheikh Mohammed Shriful Islam, Jenna McVicar, Gholamreza Askari, Mahsa Malekahmadi

**Affiliations:** 1 Department of Community Nutrition, School of Nutrition and Food Sciences, Isfahan University of Medical Sciences, Isfahan, Iran; 2 Social Development and Health Promotion Research Center, Gonabad University of Medical Sciences, Gonabad, Iran; 3 Cellular and Molecular Research Center, Research Institute for Health Development, Kurdistan University of Medical Sciences, Sanandaj, Iran; 4 Department of Epidemiology, School of Public Health and Safety, Shahid Beheshti University of Medical Sciences, Tehran, Iran; 5 Institute for Physical Activity and Nutrition (IPAN), School of Exercise and Nutrition Sciences, Deakin University, Melbourne, Australia; 6 Research Center for Gastroenterology and Liver Disease, Shahid Beheshti University of Medical Sciences, Tehran, Iran

**Correction to: BMC Pediatr 21, 178 (2021)**

**https://doi.org/10.1186/s12887-021-02631-1**

Following the publication of the original article [1], the authors found out that affiliations were assigned incorrectly. Corrected information is provided in the author group section above and the affiliation section of this article.

The original article has been corrected.
